# Multimodal Liver-Directed Management of Neuroendocrine Hepatic Metastases

**DOI:** 10.4061/2011/452343

**Published:** 2011-10-29

**Authors:** Mark A. Lewis, Joleen Hubbard

**Affiliations:** Division of Medical Oncology, Mayo Clinic, 200 First Street SW, Rochester, MN 55905, USA

## Abstract

A preponderance of patients with neuroendocrine tumors (NETs) will experience hepatic metastases during the course of their disease. Many diagnoses of NETs are made only after the neoplasms have spread from their primary gastroenteropancreatic sites to the liver. This paper reviews current evidence-based treatments for neuroendocrine hepatic metastases, encompassing surgery, hepatic artery embolization (HAE) and chemoembolization (HACE), radioembolization, hepatic artery infusion (HAI), thermal ablation (radiofrequency, microwave, and cryoablation), alcohol ablation, and liver transplantation as therapeutic modalities. Consideration of a multidisciplinary approach to liver-directed therapy is strongly encouraged to limit morbidity and mortality in this patient population.

## 1. Introduction

Once considered extremely rare, neuroendocrine tumors (NETs), which include carcinoid tumors and pancreatic islet cell tumors, are increasing in incidence [1] and prevalence and likely remain underdiagnosed [2]. The disease course of these tumors is far from universally indolent, and metastatic involvement of the liver typically represents the greatest threat of morbidity and mortality posed by these malignancies. Regardless of their site of origin in the intestine, pancreas, or elsewhere, neuroendocrine carcinomas can compromise normal hepatic function when they spread to the liver parenchyma. Furthermore, midgut carcinoids, whose secretory peptides would ordinarily have been inactivated through enterohepatic circulation, can circumvent this first-pass clearance and then release vasoactive amines into systemic bloodflow, causing the carcinoid syndrome [3]. 

Pathologically, neuroendocrine carcinomas constitute a substantial fraction of noncolorectal metastases to the liver [4], and some of the techniques honed during management of oligometastatic colorectal disease have been applied to NETs. Survival after NETs metastasize to the liver is usually longer than the median survival times encountered in stage IV colon or rectal cancer and thus may provide a wider window of opportunity for intervention. However, the unique biology of NETs means that the principles applied to colorectal metastases cannot be fully extrapolated to management of this less common tumor type. For instance, neuroendocrine metastases tend to be more numerous and hypervascular, which may affect surgical decision making and lower the threshold for ablative approaches by interventional radiology [5].

## 2. Surgery

Surgery remains the treatment of choice. Neuroendocrine metastases are often well circumscribed and are less likely to encase or invade vascular and biliary structures than other malignancies which spread to the liver parenchyma, generally improving their resectability as a histologic group [5]. The aim of hepatic metastasectomy is removal of all gross tumor [6], but it is important to attempt cytoreduction, if feasible, even when total resection is not possible [7]. In the past, bilobar involvement or concerns about high recurrence rates meant many patients were not offered excisions of their metastases. However, increasing experience with more extensive surgeries and the coupling of aggressive resections to favorable outcomes have expanded the modern criteria of operative eligibility [8]. 

Radiographic evaluation of the liver is mandatory for appropriate surgical planning. Although miliary dissemination of disease throughout the liver was considered unusual in the past [9], it is far from uncommon to encounter metastases of varying sizes in both hepatic lobes, and preoperative imaging and postoperative pathologic analysis alike can miss small lesions if the liver is not examined in sufficiently thin slices. An important study by Dromain and colleagues compared the relative sensitivities of MRI, CT, and somatostatin receptor scintigraphy (SRS, coupled to single-photon emission coupled CT) in detecting well-differentiated neuroendocrine metastases to the liver. 64 patients underwent each of these 3 imaging modalities in random sequence preprocedurally, and the pathology yielded by either liver biopsy or surgery was then compared to the radiographic findings. MRI detected 190 hepatic metastases missed by SRS and 69 missed by spiral CT. As such, liver MRI was recommended as the single most useful imaging modality in assessing the hepatic metastatic burden from NETs [10].

At least half of NET patients will have more than 50% of their liver replaced at the time metastases are first recognized [11], but the percentage of involvement of the hepatic parenchyma by tumor does not necessarily affect surgical outcome [12]. The resection of more than three segments of the liver is necessary in the majority of cases [13], with a goal of debulking 90% or more of the appreciable tumor burden [14]. In a retrospective univariate outcomes analysis by Saxena et al., of 40 patients undergoing concomitant resection and cryoablation, resection of 3 or more liver segments predicted for a poorer progression-free survival, but there was no difference in overall survival based on the number of segments resected [15]. In a separate retrospective single-institution series described by Saxena et al. of 74 patients undergoing resection of neuroendocrine metastases (of whom 38 underwent simultaneous cryotherapy), median postresection progression-free and overall survivals were reported at 23 and 95 months, respectively, with 40% postoperative 10-year survival; high histologic grade and extrahepatic disease were significantly associated with shorter survival [16]. 33 of the patients had their tumor necrosis status recorded as a binary variable; 9 of the 33 cases demonstrated tumor necrosis, which was significantly associated (*P* = .047) with poorer overall survival in univariate analysis. Suggested operative selection criteria (Table 1) thus include little to no extrahepatic disease, no indication of tumor necrosis, and well-differentiated (versus poorly differentiated) disease [15]. 

Operative planning must address the possibility of distant, nonprimary disease at the time when liver metastases are diagnosed. Among the available imaging modalities, octreotide scintigraphy may have the greatest utility in detecting or excluding extrahepatic disease [17]. This technique relies upon the binding of radiolabeled octreotide to the somatostatin receptor (especially subtype 2) expressed on the surface of the NETs in order to make tumors conspicuous during the nuclear medicine study. Pitfalls of this approach include high background uptake of the radioligand by abdominal organs, for example, the spleen, liver, kidneys, and gut lymphoid tissue, as well as variable tumor differentiation and receptor expression affecting homogenous binding of the octreotide at the site of disease [18]. There is also a lower limit of detection based upon tumor size, such that the radiolabeling most reliably highlights lesions at least 1 cm in diameter, so scintigraphy remains mostly adjunctive to other modes of disease visualization and is now seldom used without correlation to higher-resolution cross-sectional imaging [19]. A study by Montravers et al. of 30 French patients with well-differentiated NETs in the digestive tract suggested that histology should be factored into the selection of the optimal nuclear imaging modality. While they found that (111)In-pentetreotide scintigraphy did not differentially detect carcinoid versus noncarcinoid tumors (as defined by the 2000 WHO classification of NETs [20]), 18^F^-fluorodihydroxyphenylalanine (^18F^F-FDOPA) PET had a higher detection rate of carcinoid tumors, leading the authors to recommend PET over scintigraphy in the clinical contexts of detecting the primary carcinoid tumor, staging/restaging, and identifying otherwise-occult recurrences [21]. Focusing on the liver itself, Bechener et al. examined the relative utility of PET versus scintigraphy in assessing hepatic disease burden from both carcinoid and noncarcinoid NETs. In 17 patients later proven to have liver metastases, the sensitivity of ^18F^-FDOPA PET was 81.3% versus 75% with scintigraphy, offering only a fractional benefit for the purposes of surgical planning. Moreover, the specificity of PET was lower than scintigraphy (85.7% versus 100%, resp.) due to false-positive hypermetabolism at the site of a metastatic lesion that had previously been embolized [22].

Carcinomatosis is a form of extrahepatic disease that can be particularly difficult to discern preoperatively, as tumor nodules usually have to be >1 cm in size to be reliably discernible on MRI, CT, or octreotide scintigraphy. Furthermore, the abdominal discomfort related to peritoneal involvement can be highly nonspecific and indistinguishable by history from pain related to the primary tumor. Ascites is a finding that raises the index of suspicion for carcinomatosis, but negative ascitic fluid cytology does not rule out peritoneal involvement. Minimally invasive staging laparoscopy can be particularly helpful in such cases and also allow visual inspection of liver surfaces, adding to the assessment of the hepatic disease burden [23]. It should be noted that concurrent carcinomatosis is not an absolute exclusion criterion when considering patients for hepatic metastasectomy. In a multicenter French study, among 116 consecutive patients seen for digestive endocrine tumors, Vasseur et al. identified 9 patients with both liver metastases and peritoneal carcinomatosis; of the 5 deaths that occurred during their followup, 4 were related to the progression of liver metastases, and no death resulted from the peritoneal carcinomatosis. As such, the authors concluded (admittedly on the basis of a small sample size) that the presence versus absence of peritoneal involvement by the neuroendocrine malignancy did not affect survival, and that carcinomatosis should not detract from efforts to control more life-threatening liver involvement [24].

If there is concern that resection will result in insufficient residual liver volume, that is, <30% functional liver, portal vein embolization (PVE) can be performed about a month before surgery to induce hypertrophy of the parenchyma that will constitute the postoperative hepatic remnant [25]. Embolization of the right portal vein, for instance, can induce enlargement of the left hepatic lobe in cases where right hepatectomy is planned, especially if local ablative therapies will also be applied in the left lobe. The coupling of ablation (described in more detail below) to extended liver resection (±PVE) has expanded the eligibility of patients with bilobar disease to receive directed cytoreductive therapy.

In addition to preoperative imaging, closely evaluating the histologic characteristics of the NET constitutes an important step in surgical decision making. Tumor differentiation and the Ki-67 proliferative index are independent prognostic factors of survival and may influence the decision to pursue an operation. In a single-institution study of 63 consecutive patients presenting with NETs metastatic to the liver, Hentic et al. reported that the 18 patients with poorly differentiated NETs had a 5-year survival of 6%, markedly inferior to the respective 89% and 36% 5-year survival rates in cases of well-differentiated NETs with <15% and ≥15% Ki-67 indices. The median survival of the poorly differentiated cohort (who, by definition, had Ki-67 >20%) was 14 months, and only 1 of these 18 patients underwent resection (versus 15 of the 45 patients with well-differentiated NETs who proceeded to surgery). The authors concluded that, among their patients with well-differentiated NETs, Ki-67 carried more prognostic importance than the extent of liver involvement and postulated that an aggressive surgical approach likely explained the favorable survival rates in this cohort [26].

Although overall survival from NETs appears to worsen with hormonally active tumors versus their nonfunctional counterparts [1], survival after hepatic metastasectomy does not differ between functional and nonfunctional tumors [8]. While echocardiography is obviously an important component of preoperative assessment for carcinoid patients [27], it has been shown that even patients who have already progressed to carcinoid heart disease have slower declines in cardiac function and prolonged survival after resection of their hepatic metastases, so this subgroup should not be reflexively excluded from surgical consideration [28] (although perioperative consultation with cardiology is strongly advised). Indeed, NET patients with endocrinopathies may stand to experience greater symptomatic benefit and improved quality of life after their operations. 

Outcome data from several trials examining neuroendocrine hepatic metastasectomy are summarized in Table 2. A 2009 Cochrane Database review of the published literature available at that time did not identify any randomized trials to compare surgical versus nonsurgical treatment of NET metastases to the liver, nor “any quasirandomised studies, cohort studies, or case-control studies that could inform meaningfully,” but still concluded that surgery was apparently the mainstay of survival-prolonging management [29]. The tendency of the neuroendocrine tumors to reappear is a persistent problem [17] but should not create an attitude of surgical nihilism. Recurrence does not preclude the potential for initial cytoreduction to offer significant gains in symptom control and survival. The rates of recurrence seen at the site of resection may actually be lower in NETs than with other hepatic malignancies [6].

## 3. Hepatic Artery Embolization and Chemoembolization

Because liver metastases derive most of their blood supply from the hepatic artery, local devascularization offers a targeted approach that takes advantage of neoplastic hypervascularity, especially as healthy hepatocytes derive most of their blood supply from the portal vein. Selective tumor ischemia by occlusion of the hepatic artery, accomplished either through “bland” embolization using only particles of polyvinyl alcohol or through the augmented infusion of a chemotherapeutic slurry (such as doxorubicin/mitomycin) or microparticles [35], is an attractive strategy with which to diminish the NET metastatic burden and improve quality of life for patients who are not surgical candidates. The infusions are accomplished via a catheter inserted under fluoroscopic guidance into the celiac or mesenteric arteries and then advanced into the hepatic vasculature so that the interventional radiologist can select the downstream vascular territory to be embolized.

Response rates after embolization vary between 50 and 96% from study to study, depending partly upon which criteria of radiographic regression, symptom control, and/or biochemical improvement are used [36–40]. Median duration of response extends up to 18 months [5]. Low- and intermediate-grade neuroendocrine carcinomas are more likely to show a durable response to hepatic artery embolization (HAE) and chemoembolization (HACE), whereas the proliferative rate of high-grade neoplasms will usually outpace regeneration of normal hepatocytes, increasing the likelihood of recurrence and unfavorably shifting the procedure's risk:benefit ratio [41]. It is inevitable that, in spite of careful efforts to limit obstruction exclusively to the lesion's vascular supply, some normal hepatic parenchyma will be affected by embolization of even very distal vessels, and the “postembolization syndrome”—variably defined as the constellation of elevated liver function tests, right upper quadrant pain, nausea/vomiting, and fever—should be anticipated with appropriate supportive care. A practice shift away from common hepatic artery occlusion or simultaneous bilobar treatment towards sequential, lateralized embolization of the left or right hepatic artery has decreased the incidence of fulminant hepatic failure after HAE and HACE [42, 43].

## 4. Radioembolization

Radioembolization is similar in principle to chemoembolization but uses radioactive microspheres of ytrrium-90 (^90^Y) in combination with embolic agents [44]. Again, the hypervascularity of neuroendocrine metastases makes them amenable to this approach, as high-energy beta-particles can be preferentially delivered to heavily perfused tumors with relative sparing of normal liver parenchyma [41]. Theraspheres (MDS Nordion, Ottawa, ON, Canada) and SIR-Spheres (Sirtex Medical Limited, New South Wales, Australia) refer to proprietary radiopharmaceuticals that differ in the respective composition (nonbiodegradable glass versus biodegradable resin) and diameter (20–30 *μ*m versus 20–60 *μ*m) of their microspheres [44]. An MD Anderson study of 8 patients given SIR-Spheres for NETs (6 islet cell tumors, 2 carcinoids) delivered a median first radiation dose of 35.75 mCi. All 8 patients had disease that had previously been treated with HAE or HACE; the SIR-Spheres produced a partial response in 1 patient and stable disease in 4 patients, but 3 patients progressed [41]. The study provided proof of principle that the intermixture of radioactive and nonradioactive embolic agents does not preclude the possibility of response from neuroendocrine hepatic metastases, and higher response rates have been observed in other series; for instance, in a single-institution study performed upon 34 patients with nonresectable metastatic disease, King et al. described 50% or greater symptomatic and radiographic responses, with an 18% rate of complete response on imaging, and a mean overall survival of 29.4 months [45]. In the MD Anderson study, postembolization sequelae were considered more tolerable after radioembolization when compared to the after effects of HAE and HACE, but 3 patients developed abdominal pain after the first ^90^Y treatment that then prevented them from proceeding to a planned second treatment [41]. Another important distinction from HAE and HACE is that radioembolization patients should undergo preprocedural evaluation for hepatopulmonary shunts to ensure that no more than 20% of bloodflow is diverted to the lungs and to minimize extrahepatic delivery of yttrium [44]. Circulatory reflux into the gastroduodenal arteries also increases the risk of irradiation beyond target lesions in the liver. Pretherapeutic technetium-99m-(^99m^Tc-) labeled macroaggregated albumin (MAA) scans can exclude these conditions [46]. Patients who are deemed untreatable on the basis of unacceptably high arteriohepatovenous shunting can actually have their shunts occluded through the temporary inflation of balloons within the hepatic veins, which may then enable radioembolization to occur more safely [47]. The risk of radiation pneumonitis and GI toxicity associated with radioembolization should be balanced against the potential benefit of the ^90^Y beads as salvage therapy for unresectable liver disease, especially in NET patients for whom carcinoid syndrome is significantly detrimental to their quality of life [48].

## 5. Hepatic Artery Infusion

Hepatic artery infusion involves placing a pump inside the hepatic artery for the direct delivery of chemotherapeutic agents to the downstream vascular territories [44]. While this specialized technique is most often applied to the treatment of colorectal metastases, and even then only in tertiary care centers, it has rarely been used in the management of metastatic NETs, mostly as an adjunct to chemoembolization [49]. In a study by Christante et al., 77 patients with hepatic neuroendocrine metastases who progressed despite treatment with somatostatin analogues were treated either with HAI then HACE (59 patients), or with HAI alone (18 patients). The infusion regimen consisted of four monthly instillations of 5-fluorouracil. The overall response rate, measuring radiographic or symptomatic improvement, was 80%. Median progression-free survival was 19 months, with all patients initiating HAI when their hepatic disease first enlarged during octreotide therapy. However, the median disease-specific survival of 39 months was not clearly different from the outcomes of bland embolization or HACE without HAI [49]. Outcomes from various trials of intra-arterial therapy are reported in Table 3.

## 6. Thermal Ablation

Thermal ablative approaches to hepatic metastases rely on the cytotoxic effects of nonphysiologic temperatures that are focally induced within the liver by carefully placed probes. These instruments are designed to create extreme heat or cold, either of which can result in cell death, within a confined range of surrounding tissue. Radiofrequency ablation (RFA) and microwave ablation (MWA) are the most popular methods for inducing heat-related cell death [55]. Cryoablation, in contrast, produces damaging temperatures far below the freezing point of intracellular water. Ablation techniques can be applied in the setting of inoperable disease, or, at the surgeon's discretion, as a complement to resection, for example, to eradicate small foci of disease deep in the hepatic parenchyma with minimal disturbance to surrounding tissues and optimal preservation of residual liver. 

In RFA, high-frequency current courses through an electrode, which is inserted in needle-like fashion into the target lesion under ultrasound or CT guidance, through percutaneous or laparoscopic approaches, or during laparotomy [55, 56]. Heat is generated after a change in the direction of the alternating current causes ionic vibration [57]. Intracellular proteins will denature and lipid bilayers will melt after fewer than two minutes above temperatures of 60°C, and the cells through which the radiofrequency electrical current passes directly can reach temperatures above 100°C, which boils the tissue and creates water vapor [58]; lower temperatures require longer exposure times, for example, eight minutes at 46°C are needed to induce coagulative necrosis of malignant cells through thrombosis of their microvasculature [58]. Beyond the zone of complete coagulation, tissue will be partly destroyed in a spherical distribution up to 0.8 cm in diameter. A single electrode can induce cell death up to 1.6 cm from the center of the tumor [59], and multiple electrodes can be deployed in an array to create a spherical burn beyond 5 cm in diameter [60]. Intraprocedural ultrasound can almost immediately assess the size of the necrotic zone to maximize the likelihood of adequate thermal damage to the site of known metastasis, which should, in theory, lower recurrence rates [61].

One of the largest prospective trials of RFA, which included 54 patients with unresectable hepatic metastases from carcinoid or islet cell tumors (as well as 9 patients with metastases from medullary thyroid carcinoma), and in which ablation was performed laparoscopically under ultrasound guidance, demonstrated a median survival of 3.9 years following the first ablation, and extrahepatic disease was not a criterion for exclusion. In measuring the diameter of the largest liver lesion targeted for ablation, 3 centimeters was an important cutpoint for predicting survival, with patients whose dominant lesions were at or above this threshold experiencing a median survival fewer than 3 years, whereas the median survival for patients with dominant lesions smaller than 3 centimeters had not been reached by the time the study concluded. Over 90% of patients reported postablation symptomatic improvement, and the median duration of symptom control was 11 months. Male gender was also significantly associated with poorer postablation survival, for reasons that were unclear [62]. 

In an even larger study, Mulier et al. performed a meta-analysis of 95 separate series describing the use of RFA in the control of liver tumors. The pooled data allowed the authors to examine the postablation outcomes of 5224 hepatic lesions of varying histologies. 11 of the 330 reported neuroendocrine metastases demonstrated recurrence, with a minimum follow-up period of 6 months, and this 3.3% rate of recurrence for NETs was the lowest among the different categories of pathology, that is, versus hepatocellular carcinoma and metastases from colon, breast, and unspecified primary tumors. Indeed, in univariate analysis, neuroendocrine histology was a tumor-dependent factor significantly associated with a lower likelihood of local recurrence, along with smaller size (<3 cm versus 3–5 cm and >5 cm), a nonsubcapsular (versus subcapsular) location, and distance from a major vessel. When these factors were subjected to multivariate analysis, however, only a small size of the ablated lesion was significantly associated with lower recurrence, with Mulier and colleagues commenting that the minimum postablation followup of 6 months may have been a too short interval and thus underestimated the recurrence rate in NETs with a slower mean natural growth rate [63].

Microwaves are a nonionizing form of radiation that causes extremely rapid oscillation of the water within tissues, with dipolar reversals occurring a billion times per second. Friction from the fluctuation of intracellular water molecules generates heat, which in turn leads to coagulative necrosis [64]. The intratumoral temperatures of MWA are consistently higher than can be achieved with RFA [65]. Also, as opposed to the mostly passive conduction of heat in RFA, in which the “heat sink” effect of relatively cool nearby bloodflow can result in incomplete ablation of tumors close to the larger hepatic vessels, the MWA method involves active heating that may be more appropriate for targeting tumor sites next to major hepatic vasculature [55, 64]. As in RFA, a probe is placed into the target lesion under radiographic guidance or during open surgery. Multiple lesions can be ablated during the same procedure. The clinical experience with MWA has, to date, mostly involved treatment of hepatocellular carcinoma, but neuroendocrine tumors have been included in some series. Martin et al. described that, of 100 patients undergoing MWA for primary or secondary hepatic tumors at their institution during a 5-year period, 11 had neuroendocrine pathology. A 90% success rate for complete ablation was reported for carcinoid tumors, with no recurrences at the ablation sites. The authors noted that the multiplicity of lesions in metastatic carcinoid, as well as the intraprocedural difficulties of locating all the tumors seen on preprocedural CT, prevented achievement of a 100% complete ablation rate [65]. It is important to recognize that the majority of these patients had MWA performed under ultrasound guidance during open surgery, that is, concomitant hepatectomy and/or extrahepatic metastasectomy [65]. There is still a paucity of data comparing MWA (especially performed percutaneously) to RFA for management of NETs in the liver. 

Cryoablation is the most mature thermoablation technique, having first been proposed in 1851 [66]. Cell viability is decreased at low temperatures, depending partly on the rate of cooling and the spatial relationship to ice formation around the cryoablation probe [67]. While cryogenic temperatures can both preserve and destroy tissue [68], the primary determinant of cell death is the depth of the lowest obtained tissue temperature, which should be −50°C to achieve necrosis in neoplastic tissue [69]. In addition to near-immediate mechanical injury caused during a freeze by ice crystals disrupting their membranes and organelles, cells can die during the thaw and postthaw periods due to disrupted vascular supply or due to cold-activated endonucleases triggering an apoptotic response [68]. However, malignant cells may be more resistant to lethal damage from freezing compared to hyperthermia [58], and some studies have reported higher complication and recurrence rates when cryoablation is compared against heat-based therapies [55]. Seifert et al. described a series of 13 patients with NETs who underwent hepatic cryotherapy; in each case, the cryoprobes were inserted under ultrasound guidance, and freezing was monitored until the ball of ice extended beyond the tumor for 1 cm in all directions. 12 of 13 patients had complete ablation of all visible tumors, with 2 recurrences at the ablation sites and 12 survivors at 1 year of followup. All 7 patients who had hormonally related symptoms prior to cryotherapy experienced palliative benefit. 2 patients developed a postprocedural coagulopathy requiring intra-abdominal packing and the transfusion of clotting factors. The authors had not observed similar bleeding complications when applying their cryosurgical techniques to patients with hepatocellular carcinoma and speculated that the necrosing carcinoid tumors were releasing substances into circulation that disrupted the coagulation cascade. All patients demonstrated thrombocytopenia two days after the procedure [70]. In a larger series by Bilchik et al. of 17 patients undergoing hepatic cryosurgery for NETs, all patients demonstrated a transient coagulopathy, requiring transfusion of either platelets or fresh frozen plasma (with an average infusion of 4 units per procedure) [66]. 

Outcomes for various studies of ablative therapies in the setting of NETs metastatic to the liver are summarized in Table 4.

## 7. Alcohol Ablation

Ultrasound-guided injection of ethanol, otherwise known as percutaneous alcohol injection (PAI), into neuroendocrine metastases has been described in multiple series [75^,^ 76], none of which were histologically exclusive to NETs. A 1994 report by Giovannini and Sietz included 5 NETs among 40 patients with various pathologies undergoing PAI, and complete necrosis rates in carcinoid tumors were inferior to the responses seen in colorectal metastases [73]. Nonetheless, PAI could be an advantageous technique over RFA when tumors are located next to large vessels that would be vulnerable to the “heat sink” effect, or in proximity to central bile ducts that tend to stricture in response to heat [5]. Lesions chosen for ethanol ablation are necessarily less than 5 cm in diameter and the cubic volume of alcohol injected requires modeling the target tumor as a sphere, but these estimations are more likely to be accurate when the radius is shorter, and very small metastases can be ablated with minimal collateral damage to the surrounding liver [5]. In a smaller study by Livraghi et al., in which 2 of 14 patients with liver metastases had neuroendocrine carcinoma, the patients demonstrated a complete response after ablation of 4 lesions smaller than 3.1 cm [74]. Thus, PAI is best used not as monotherapy but rather as an adjunct to newer ablative techniques when approaching tiny or inauspiciously located metastases [5, 75]. All of the ablation methods are summarized in Table 5. 

## 8. Liver Transplantation

On its list of indications for orthotopic liver transplantation, the United Network for Organ Sharing (UNOS) includes “metastatic neuroendocrine tumors (carcinoid tumors, APUDomas, gastrinomas, glucagonomas) in persons with severe symptoms and with metastases restricted to the liver, who are unresponsive to adjuvant therapy after aggressive surgical resection including excision of the primary lesion and reduction of hepatic metastases.” However, the published experience with liver transplantation for NETs remains limited, and fewer than 300 unique cases are described in the literature [80]. A meta-analysis of 20 studies encompassing 89 patients transplanted for metastatic pancreatic NETs reported cumulative 1-, 3-, and 5-year survival rates of 71%, 55%, and 44%, respectively. Recurrence-free survivals were 84%, 47%, and 47% at the same respective time points. If patients were 55 years old or younger and were not undergoing simultaneous pancreatic resection, then their predicted 5-year survival was 61%. Conversely, a 0% 5-year survival rate was prognosticated for patients older than 55 who were undergoing resection of the primary pancreatic lesion at the same time as transplantation [77]. The largest single-center experience with liver transplantation for NETs reports a 10-year survival rate of 50% among 19 patients. 12 patients recurred, within 2 weeks to 48 months from the date of transplant. 3 patients had recurrence-free survivals beyond 8 years. 7-year survival was 100% in the 5 patients with Ki67 in less than 5% of their tumor cells and normal expression of E-cadherin, that is, positive membrane and absent cytoplasmic staining [78]. A multicenter French study pooled 85 cases of liver transplantation performed for NETs between 1989 and 2005. 34 of the patients underwent concurrent resection of extrahepatic disease, which in 7 cases required upper abdominal exenteration (resection of the pancreas, spleen, stomach, and duodenum, with 3 patients receiving en bloc composite liver-duodenum-pancreas grafts). Concomitant exenteration had the strongest association with death in multivariate analysis (RR: 3.72, 95% CI: 1.54–8.95, *P* = .0034), with 0% 3-year survival and a median survival of 1.5 months, whereas the median survival among all patients was 56 months. Excluding exenteration, the most important prognostic factors were a duodenopancreatic location of the primary tumor and hepatomegaly (≥120% standard liver volume in the explanted organ); the 23 patients with both of these attributes had a 12% 5-year survival versus 68% 5-year survival among the 55 patients with only one or neither of these factors [79]. Clearly, the decision to transplant a patient with neuroendocrine carcinoma metastatic to the liver requires careful consideration of numerous clinicopathologic variables, and mortality is higher in older individuals requiring concurrent disease resections. Outcomes for studies of liver transplantation for involvement by metastatic NETs are summarized in Table 6.

## 9. Conclusion

**Figure 1:**
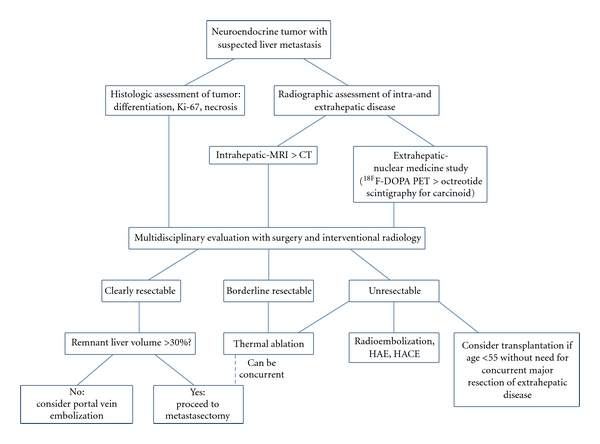
Liver-directed treatment algorithm for neuroendocrine hepatic metastases.

The unique tumor biology of neuroendocrine carcinomas presents disease-specific challenges when hepatic metastases occur [81], but some characteristics of these neoplasms lend themselves to management with liver-directed therapy. Medical oncologists should work in multidisciplinary fashion with surgeons and interventional radiologists to assess the potential utility of these organ-focused techniques in series with exciting advances in systemic management of NETs, such as peptide receptor radionuclide therapy (PRRT) [82] and promising chemotherapeutic agents like sunitinib [83] and everolimus [84]. It is beyond the scope of this review of liver-directed therapy to adequately address PRRT and chemotherapy, but, in conclusion, we propose the algorithm shown in (Figure 1) for approaching neuroendocrine hepatic metastases.

## Figures and Tables

**Table 1:** Suggested eligibility criteria for resection of NET liver metastases.

No miliary disease on preoperative liver imaging(MRI or multidetector CT)
Little to no extrahepatic disease on preoperative nuclear medicine studies
(^18F^F-DOPA PET preferred over octreotide scintigraphy for carcinoid)

Well- or moderately differentiated neuroendocrine carcinoma
(Ki-67 <20% and ideally, <15%)

Projected volume of residual functional liver >30**%**

No tumor necrosis

**Table 2:** Summary of outcomes from resection of neuroendocrine liver metastases. OS: overall survival; PFS: progression-free survival.

First author, publication year	No. of surgical patients	Median followup, months	Survival data	Predictors of survival
Mayo, 2011 [30]	339 [66 with simultaneous ablation]	26	Median OS: 123 months 5-year survival: 74%	Symptomatic high-volume [>25% liver involved] disease benefited most from surgery (versus intra-arterial therapy, *P* < .001)
Saxena, 2011 [16]	74 [38 with simultaneous cryoablation]	41	Median PFS: 23 months	Worse PFS with R1 (versus R0) pathologic margin status (*P* = .023)
			Median OS: 95 months	Worse OS from higher grade (well versus moderate versus poor differentiation, *P* < .001) and extrahepatic disease (*P* = .021)
Karabulut, 2011 [31]	27 [excluding subsequent liver transplants]	29	Median PFS: 15 months Median OS: 190 months	Margin status did not affect OS; in outcomes analysis including RFA and embolization, worse OS with male gender (*P* = .04), dominant metastasis >5 cm (versus <3 cm, *P* = .04), extrahepatic disease (*P* = .03)
Glazer, 2010 [32]	172 [120 with small bowel or pancreatic primaries; 18 had only RFA]	50	Median OS: 116 months 5-year survival: 77.4% 10-year survival: 50.4%	Increasing time interval from primary resection to hepatic metastases predicted for poorer survival (*P* = .01)
Sarmiento, 2003 [8]	170 [75 with complete resection]	Not reported (excluded <12 months followup)	Median OS: 81 months 5-year survival: 61% 10-year survival: 35%	No OS difference with or without endocrinopathy (60% versus 61% at 5 years, *P* = .75), no OS difference between carcinoid and islet cell (87 versus 66 months, *P* = .058)
Elias, 2003 [33]	47 [36 with concurrent extrahepatic resection]	62	Median OS: 91 months 5-year survival: 71% 10-year survival: 35%	Worse DFS with incomplete surgery (R2 versus R1 versus R0, *P* = .003), pancreatic origin (*P* = .01), bilateral liver involvement (*P* = .01); no factor predicted OS
Chen, 1998 [34]	15	27	5-year survival: 73% [versus 29% in 23 patients with unresectable disease]	Median survival not reached in resection group, but OS significantly longer than unresected (*P* = .003)

**Table 3:** Summary of outcomes for intraarterial therapy of neuroendocrine liver metastases. HAE: hepatic artery embolization; HACE: hepatic artery chemoembolization; HAI: hepatic artery infusion; OS: overall survival.

First author, publication year	No. of embolized patients	Survival data	Comments
Paprottka, 2011 [50]	42 [^90^Y radio-embolization]	40 of 42 patients alive with mean followup of 16.2 months	36 of 38 symptomatic patients had clinical improvement within 3 months
Dong, 2010 [51]	123 [HACE]	Mean OS: 39.6 months 5-year OS: 36% 10-year OS: 20%	Baseline albumin <3.5 g/dL was a multivariate predictor for poorer OS (*P* = .003)
Kennedy, 2008 [52]	148 [^90^Y radio-embolization]	Median OS: 70 months	No radiation-induced liver disease or failure, even with retreatment
Christante, 2008 [49]	77 [18 HAI alone, 59 HAI + HACE]	Median OS [HAI alone]: 26 months Median OS [HAI + HACE]: 39 months	All 10 patients with nonfunctional neoplasms and 15 of 16 patients with islet cell neoplasms died within 5 years
Strosberg, 2006 [53]	84 [HAE]	Median OS: 36 months	Fewer symptoms in 44 of 55 patients
Gupta, 2005 [54]	123 [74 HAE, 49 HACE]	Median OS [carcinoid]: 33.8 months Median OS [islet cell]: 23.2 months	Male gender predicted worse OS (*P* = .05) for carcinoid, bone mets predicted worse OS for islet cell (*P* = .03)

**Table 4:** Summary of outcomes for ablation of neuroendocrine liver metastases. DFS: disease-free survival; MWA: microwave ablation; NET: neuroendocrine tumor; OS: overall survival; PFS: progression-free survival; RFA: radiofrequency ablation.

Author, publication year	No. of ablated patients	Median followup, months	Survival data	Comments
Karabulut, 2011 [31]	68 [RFA]	22	Median PFS: 10.5 months Median OS: 73 months	No significant overall survival difference between RFA and resection
Akylidiz, 2010 [71]	89 [RFA; 78 with NETs of GI origin, 11 medullary thyroid cancer]	30	Median DFS: 15.6 months Median OS: 72 months	Liver tumor volume (>76 cc versus <30 cc, *P* = .04), symptoms (present versus absent, *P* = .04), extrahepatic disease (*P* = .02)
Martin, 2010 [65]	11 [MWA; 7 with concomitant hepatectomy; 6 with concomitant extrahepatic resection]	36	Median DFS: 8 months Median OS: 18 months	Zero recurrences at ablation site
Mazzaglia, 2007 [62]	63 [RFA; 24 with extrahepatic disease at time of 1st ablation; 9 patients with medullary thyroid cancer]	34	Median OS: 47 months after 1st RFA 5-year survival: 48%	Male gender [3x mortality risk of female] (*P* = .04), largest tumor >3 cm (*P* = .03)
Seifert, 1998 [70]	13 [cryoablation]	13.5	12 patients alive at the end of followup (up to 103 months)	All 7 symptomatic patients had subjective improvement
Shapiro, 1998 [72]	5 [cryoablation]	30	1-year survival: 60% 2-year survival: 40%	All 5 patients had relief of carcinoid syndrome

**Table 5:** Summary of liver-directed ablation modalities.

Ablation technique	Mechanism of tumor injury	Maximum size of target lesion	Comments/caveats
RFA	Heat	1.6 cm: single electrode 5 cm: array	Prone to heat sink from adjacent vessel, ↓ control for lesions >4 cm
MWA	Heat	2 cm: single needle 4 cm: parallel needles	Less prone to heat sink, but fewer supportive data than RFA
Cryoablation	Cold	4 cm: single needle 6 cm: multiple needles	↓ control for lesions >4 cm, risk of ↓ platelets and coagulopathy
Alcohol	Toxic	4 cm	Adjunctive only

**Table 6:** Summary of outcomes for liver transplantation for neuroendocrine metastases. OS: overall survival.

Author, publication year	No. of liver transplant (LT) patients	Survival data	Predictors of survival
Gedaly, 2011 [76]	150 [13 receiving another organ at time of LT]	49% 5-year survival [excluding multiple organ transplants]	Regardless of age, improved survival (>60% at 5 years) for patients waiting more than 2 months for transplant (*P* = .005)
Mathe, 2011 [77]	89	44% 5-year survival	Worse survival with recipient age >55 (*P* = .0242) and simultaneous LT-pancreas resection (*P* = .0132)
Rosenau, 2002 [78]	19	80% 5-year survival 50% 10-year survival	Ki-67 <5% and normal E-cadherin expression had 100% 7-year survival (versus 0% when Ki-67% >5% or aberrant E-cadherin expression, *P* = .007)
Le Treut, 2008 [79]	85 [34 with concurrent extrahepatic resection]	Median OS: 56 months	Exenteration (*P* = .0034), a duodenopancreatic primary (*P* = .0018), and hepatomegaly (*P* = .0157), all predicted for poorer survival
